# Standard-free single magnetic bead evaluation: a stable nanoplatform for prostate disease differentiation†

**DOI:** 10.1039/d2sc00928e

**Published:** 2022-04-29

**Authors:** Zili Huang, Xiaobo Xie, Bei Xu, Rui Liu, Jianyu Hu, Yi Lv

**Affiliations:** Key Laboratory of Green Chemistry and Technology of Ministry of Education, College of Chemistry, Sichuan University Chengdu 610064 PR China lvy@scu.edu.cn; Analytical & Testing Center, Sichuan University Chengdu 610064 PR China; Department of Clinical Laboratory, Mianyang Central Hospital, School of Medicine, University of Electronic Science and Technology of China Mianyang 621000 PR China; Division of Analytical and Environmental Toxicology, Department of Laboratory Medicine and Pathology, Faculty of Medicine & Dentistry, University of Alberta Edmonton Alberta T6G 2G3 Canada

## Abstract

Explicit interpretation of heterogeneity between prostate-specific antigen (PSA) subtypes is essential for prostate cancer differentiation during different disease courses, whereas a universal protocol with uniform criteria is still lacking across the globe. In this work, a standard-free single magnetic bead (SMB) nanoplatform utilizing metal nanoparticles with optimal diameters was proposed for prostate disease differentiation in a 134-donor model. The inaccuracy of detection in absolute quantification was diminished *via* evaluations of metal intensities on the single magnetic bead. The intrinsic proportion of fPSA in tPSA was successfully evaluated by direct use of the Pt to Au intensity ratio (Pt/Au ratio), exhibiting better differentiation between healthy and unhealthy, benign prostatic hyperplasia (BPH) and cancer individuals compared with solo fPSA or tPSA. We generated thresholds respectively for prostate disease differentiation, envisioning that this standard-free SMB nanoplatform would establish a standardized methodology with uniform criteria worldwide in cancer diagnosis, staging, and postoperative assessments.

## Abstract 2

A standard-free stable single magnetic bead nanoplatform was proposed in this work. The use of metal signal ratio was directly applied for intrinsic biological fPSA to tPSA ratio evaluations for prostate disease differentiation.

## Graphical abstract

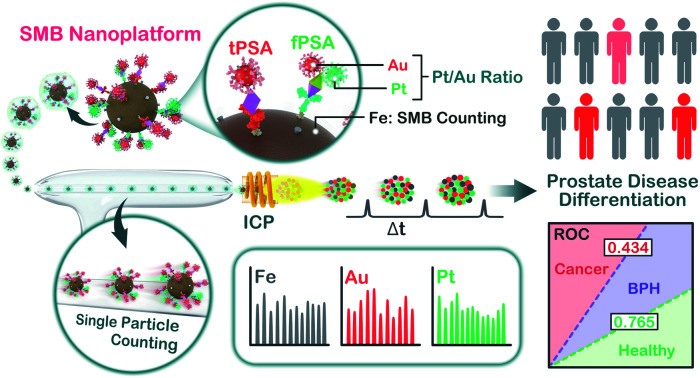

## Introduction

Precise differentiation and diagnosis of prostate cancer is necessary to unveil the different molecular expressions during varying stages of this second most frequent malignancy among men worldwide.^1–3^ Although regarded as the first-line test for prostate cancer diagnostics, total PSA (tPSA) screening appears to be controversial with high false-positive outcomes. Due to the high heterogeneity of PSA molecular subtypes, elevated levels of serum tPSA are also found in benign prostate diseases.^4^ An explicit revelation of the PSA subtype proportion during different prostate cancer stages is undoubtedly of great significance.^5,6^ Clinically, the free to total PSA ratio (% fPSA) in serum is reported to correlate with the severity of prostate disease, but reliable evaluations are challenged by two situations. One is the separate solo measurements of fPSA and tPSA for % fPSA calculation which may provide discordant results and misinterpretations of prostate conditions.^4,7^ The other is the lack of gold-normalized protocols which result in biased clinical criterion settings among users or labs across the globe.^8^ Standardized methodologies with uniform outputs are never out of date to bring simultaneous evaluations of fPSA, tPSA, and % fPSA.

Absolute quantification of these biomarkers in a single sample is always required for accurate diagnostics.^9–14^ However, the absolute intensity of biomarkers obtained from the total probe concentration can be affected by physical factors such as poor washouts or uneven bindings, possibly giving rise to inaccurate readouts.^15,16^ Moreover, specific standards are still required since the stoichiometry of the total probe concentration is hard to precisely determine.^17^ As a solution, a standard-free strategy where the intrinsic proportion of biomarker subtypes can be revealed directly by the ratio of probe signals, attracts much attention. The construction of a stable data acquisitions and processing platform with unitary criteria is an ever-increasing demand.

Endowed with high throughput and resolution, single particle ICP-MS (sp-ICP-MS) has gained great attention in multiplex biological practice and single-cell analysis.^18–24^ Based on the theory in a typical sp-ICP-MS analysis, nanoparticles are injected one by one into the detector where only one nanoparticle is measured during each reading period. Benefiting from ample metal content in one nanoparticle, sensitivity is largely improved in heterogeneous bioassays where absolute quantifications can be easily achieved by measuring the total numbers of target-specific nanoparticles.^25–27^ In addition, sp-ICP-MS allows evaluations of controllable nanoparticle aggregates by target recognition, realizing homogeneous bioassays without washing or physical separations.^28–31^ In data acquisition and processing, sp-ICP-MS focuses on each measured nanoparticle and provides the intensity calculated by the uniform distribution of transient signals.^32^ More intriguingly, the calculated intensity of nanoparticles is relatively stable and is not affected by the nanoparticle concentration.^20,21,33–35^ In other words, the inaccuracy of detection in absolute quantification can be potentially diminished even if the total probe concentration is fluctuating in parallel samples. If this unique advantage can be reasonably adopted with a standard-free strategy and multiplex bioassay, developing a standardized and universal platform with stable outcomes becomes possible.

**Fig. 1:**
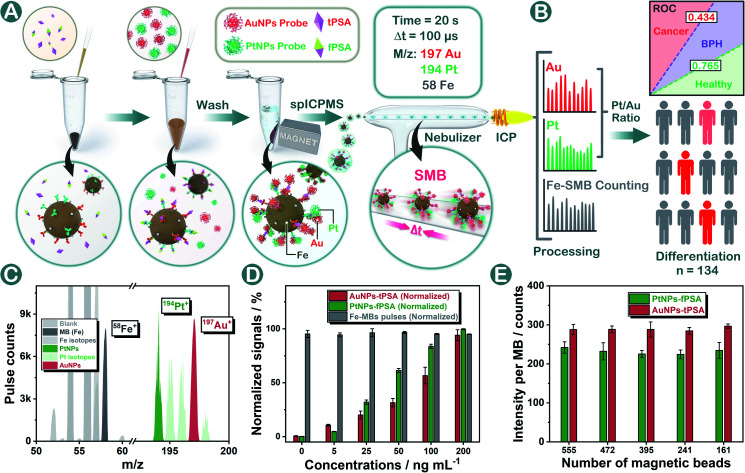
(A) and (B) schematic illustration of the proposed SMB nanoplatform for prostate disease differentiation. (C) Mass scanning of magnetic beads, AuNPs, and PtNPs. (D) Dual-biomarker evaluation using the SMB nanoplatform with the same number of magnetic beads. (E) Stable data processing of the SMB nanoplatform with varied numbers of magnetic beads.

Herein for the first time, a standard-free nanoplatform named a single magnetic bead (SMB) nanoplatform was proposed for prostate disease differentiation. In the methodology shown in Fig. 1A and B, AuNPs and PtNPs were applied as probes for tPSA and fPSA capturing on magnetic beads respectively. In our design, by controlling the same number of magnetic beads within each measurement, the SMB nanoplatform could be centered on a single magnetic bead where Au or Pt contents were calculated respectively based on Poisson intensity distributions. The metal isotopic intensity on each magnetic bead was stable and was only affected by the number of noble metal nanoparticles bound on a single magnetic bead instead of magnetic bead numbers or sporadic abnormally large spikes during the assay. The inaccuracy from fluctuating total probe concentrations caused by physical factors was efficiently diminished, guaranteeing stable outcomes for consistent bioassays. Under optimal conditions, AuNPs and PtNPs with suitable diameters achieved five order-of-magnitude linear ranges and low limits of detection (LODs) in dual-biomarker immunoassay. In prostate disease differentiation, the intrinsical biological proportion of fPSA and tPSA could be directly evaluated by using the Pt/Au intensity ratio. We generated thresholds for judging healthy or unhealthy, benign prostatic hyperplasia (BPH) or cancer respectively by using the Pt/Au ratio, hoping to establish universal criteria for cancer diagnosis without specific standards.

## Results and discussion

### Initial construction of the SMB nanoplatform

**Fig. 2:**
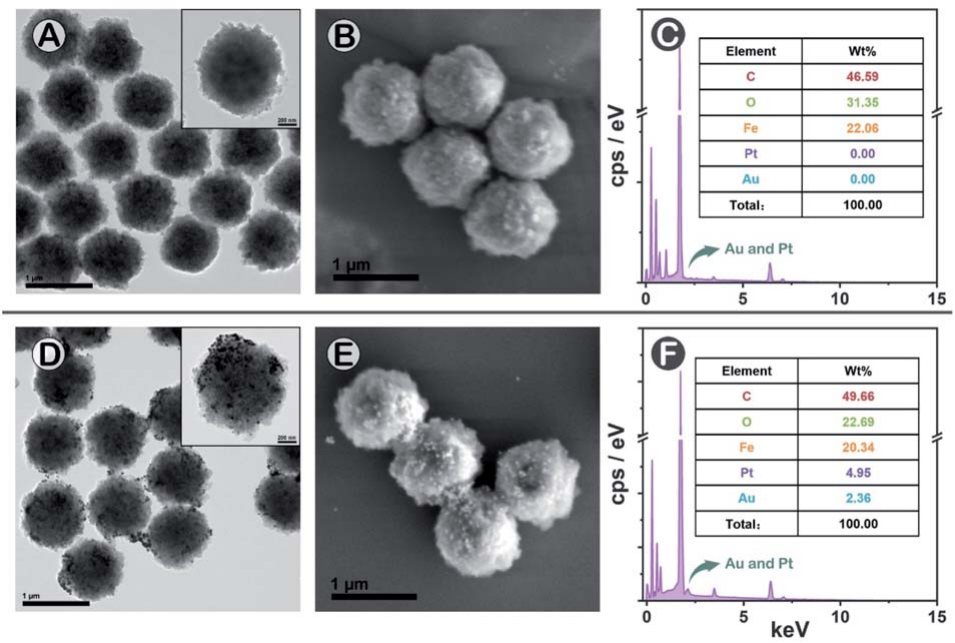
Characterization of the dual-biomarker immunoassay using (A) (blank) and (D) TEM, (B) (blank) and (E) SEM, as well as (C) (blank) and (F) EDS. The concentrations of fPSA and tPSA were 100 ng mL^−1^.

To construct the SMB nanoplatform, two noble metal nanoparticles, *i.e.*, AuNPs and PtNPs were used as biomarkers probes. As expected, the most abundant isotopic ions ^194^Pt^+^ and ^197^Au^+^ can be detected with high sensitivity without any interferences as shown in Fig. 1C. The magnetic beads as the center for capturing PtNPs or AuNPs in the presence of the target biomarker can be readily quantified by measuring the frequency of the corresponding ^58^Fe^+^ transient signals. In element mass analysis, Fe^+^ isotopes are easily affected by Ar- and Ca-based ions, therefore requiring the use of kinetic energy discrimination (KED) mode to reduce the interferences.^36^ According to the results shown in Fig. S1,† low-content ^58^Fe^+^ (∼0.3%) applied in this work exhibited distinct spike pulses which linearized with the number of magnetic beads, averting the common use of KED mode which could reduce signal sensitivity. In the presence of fPSA and tPSA, the corresponding PtNP and AuNP probes could efficiently bind to the surfaces of the magnetic beads due to the sandwich immunosorbent. The resulting sandwich structure was clearly observed by TEM, elemental mapping (Fig. 2A, D and S2†), SEM (Fig. 2B and E), and energy dispersive spectroscopy (EDS, Fig. 2C and F), as solid evidence for a successful immunoassay. The detection of fPSA or tPSA can be achieved by detecting ^197^Au^+^ and ^194^Pt^+^ with the optimal numbers of magnetic beads (working conditions of sp-ICP-MS are described in Table S1†). In the subsequent dual-biomarker immunoassay, PtNPs and AuNPs were applied for antigen capturing with the same number of magnetic beads per assay. In accordance with the results shown in Fig. 1D, the intensities of ^197^Au^+^ and ^194^Pt^+^ on a single magnetic bead increased with elevated concentrations of fPSA and tPSA.

In a typical absolute quantification, the total concentration of probes captured by all magnetic beads should be precisely measured to reduce fluctuating readouts.^37^ To evaluate the stability of this SMB nanoplatform, we changed the number of magnetic beads after immunization in each measurement and evaluated the average Au or Pt intensity on a single magnetic bead by using metal distributions (calculation formula is given in the Statistical Analysis part in the ESI†). According to the results shown in Fig. 1E, metal intensities on a single magnetic bead remained stable when the numbers of magnetic beads were varied. The reason is that changes of measured magnetic bead numbers have no effect on the numbers of PtNPs or AuNPs on a single magnetic bead by immune recognition. Signal inaccuracy during absolute quantification in sample measurement was diminished efficiently by SMB evaluation.

### Size screening by diameter-regulation immunoassays

**Fig. 3:**
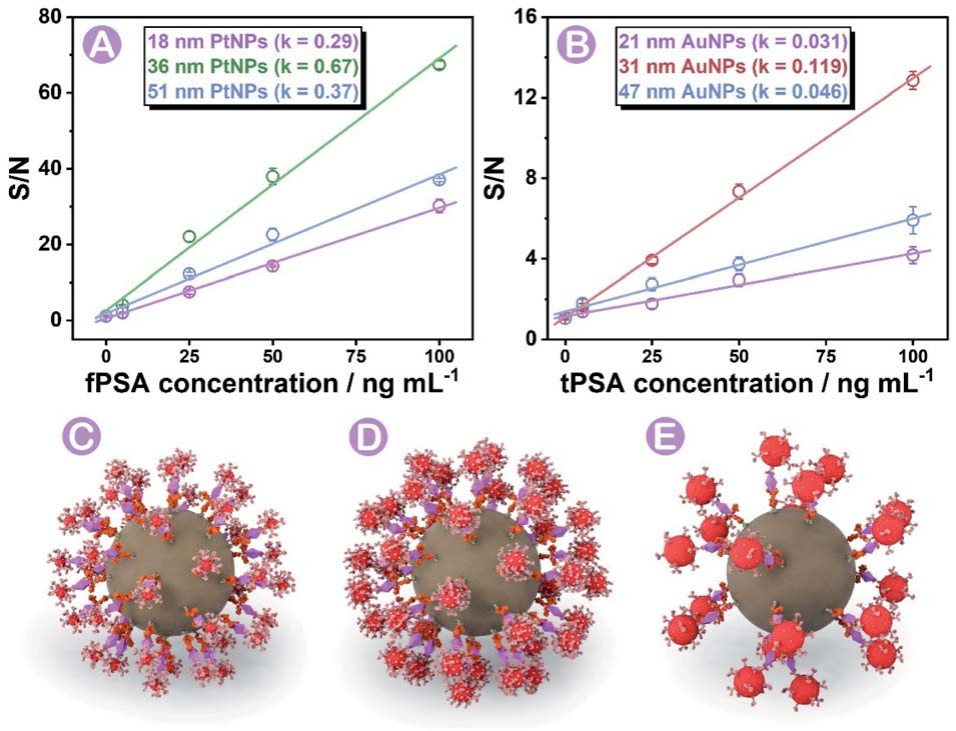
Efficiency of diameter-regulation immunoassays using PtNPs (A) and AuNPs (B) with three different diameters using the S/N ratio. Simulation of the immunoassay using AuNPs with a small (C), suitable (D), and large (E) diameter.

Based on the experience in our previous work,^28,29^ the diameter (size) of nanoparticle probes plays a crucial role in immunoassay efficiency. In this work, three different sizes of PtNPs and AuNPs (characterization studies are shown in Fig. S3, S4, and S5†) were synthesized and screened for efficient immunoassays under the same conditions (more details and nanoparticle labeling are shown in the Protocols part, Fig. S6, S8 and Table S3†). Probes affording the highest signal-to-noise (S/N) ratio were finally chosen for subsequent analysis. As shown in Fig. 3A and B, 36 ± 2 nm PtNPs and 31 ± 3 nm AuNPs exhibited the highest S/N ratio compared with relatively bigger or smaller ones. The key factors that influenced the efficiency are discussed as follows.

The size of nanoparticle probes can affect the S/N ratio of signals on each magnetic bead. As concluded in former articles,^38^ the relationship between the diameter (*d*) and intensity can be concluded as Formula a (*K* is a constant, detailed conversion of formulae is summarized in Fig. S7†) where the intensity is proportional to *d*^3^.

a *I* = *K* × *d*^3^

On one hand, the relationship between the nanoparticle size and intensity fitted to the above Formula (a), so the increase of blank signals was larger than that of the nanoparticle diameter (Fig. S7†). On the other hand, we did find that larger diameter-nanoparticle probes could cause bigger signal changes in immunoassay, but the S/N ratios did not increase as expected. AuNPs and PtNPs with a diameter around 20 nm did not cause as many intensity changes as nanoparticles with diameters of 31 and 36 nm. Combined with previous reports,^39,40^ we concluded and simulated (using AuNPs as the model) in Fig. 3C–E, that in a sphere centered SMB nanoplatform, greater steric resistance occurred and reduced the immunoassay efficiency on the magnetic bead surface when employing larger diameter-nanoparticle probes (Fig. 3E). Nanoparticles with smaller diameters (Fig. 3C) could not cause enough intensity changes. Combined with these results, 31 to 36 nm was chosen as the most suitable diameter with relatively low blank signals and high immunoassay efficiency.

### Dual-biomarker immunoassay

**Fig. 4:**
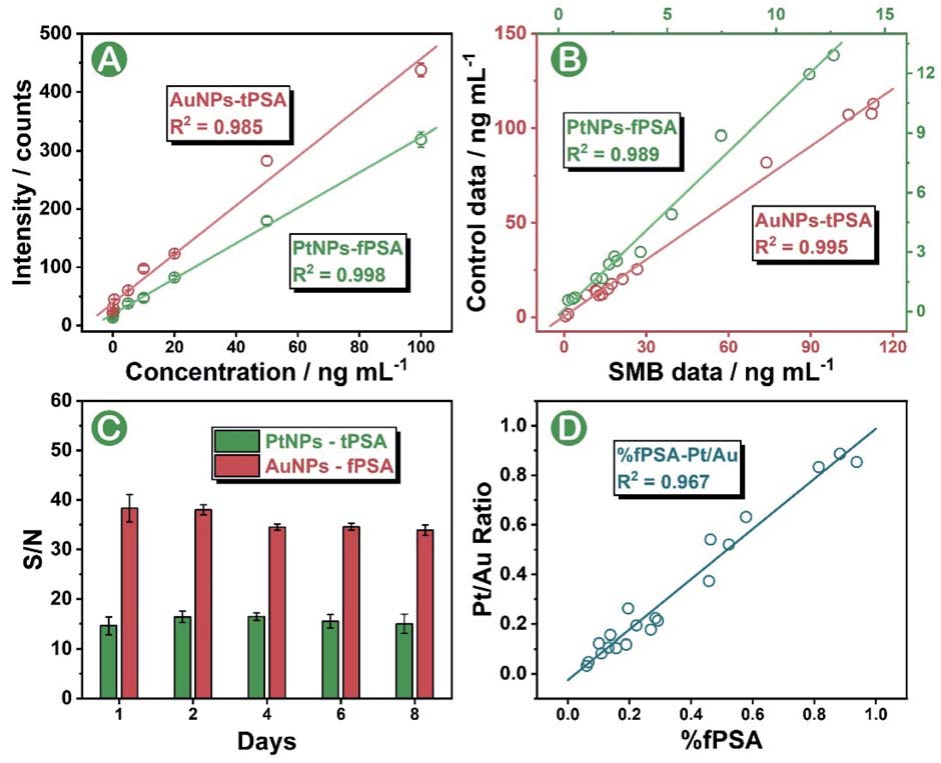
(A) SMB nanoplatform for simultaneous detection of fPSA and tPSA. (B) Data comparison between samples measured by using the SMB nanoplatform and samples with known concentrations from the hospital. (C) Stability of the SMB nanoplatform over 8 days. (D) Consistency between % fPSA and the Pt/Au ratio in 20 patients.

The biological proportion between PSA subtypes is of great value for accurate differentiation and diagnosis of prostate cancer from noncancerous prostate disease. In this work, we sought to provide a stable standard-free strategy that directly evaluated the intrinsic proportion of fPSA in tPSA for the interpretation of prostate conditions. Prior to serum sample analysis, the feasibility of the SMB nanoplatform was tested by using standard curves as well as standard addition recovery under optimal conditions (optimizations of the SMB nanoplatform is shown in Fig. S9 and S10†). After effective immune recognition, magnetic beads were washed two times and were immediately diluted for sp-ICP-MS analysis within 30 to 60 min. As shown in Fig. S11 and S12,† increased transient spikes of either ^194^Pt^+^ or ^197^Au^+^ were detected in the SMB nanoplatform at elevated fPSA or tPSA concentrations. Also, from the frequency distribution of Pt and Au intensities on a single magnetic bead (Fig. S13†), the higher the number of antigens in the sample, the higher the number of bigger-metal-intensity magnetic beads that occurred. Scrutinizing the results, 50 pg mL^−1^ to 100 ng mL^−1^ for both fPSA and tPSA were obtained from the standard curves, endowing the SMB platform with five orders-of-magnitude wide ranges (Fig. 4A). The LODs (*n* = 11) were 12 pg mL^−1^ for fPSA and 21 pg mL^−1^ for tPSA respectively which were comparable with or better than other cutting-edge strategies (shown in Table S4†) and the recommended threshold (about 3 ng mL^−1^ for tPSA and 1 ng mL^−1^ for fPSA).^8^ Combined with the recovery shown in Table S2,† the SMB nanoplatform exhibited good feasibility and sensitivity for further serum evaluations. To validate the accuracy of serum sample measurements, 15 individuals with known concentrations of fPSA and tPSA from the hospital were evaluated by using the SMB nanoplatform. Good correlations between the results and control data were found as shown in Fig. 4B. We also applied the same batch of nanoparticle probes for consecutive diagnosis of 50 ng mL^−1^ fPSA and tPSA (Fig. 4C), proving that the SMB nanoplatform could remain stable over 8 days. The consistency between the Pt to Au intensity ratio (Pt/Au ratio) and calculated % fPSA is essential for subsequent standard-free analysis. As shown in Fig. 4D, the Pt/Au ratio showed good correlations with % fPSA (calculated by using fPSA and tPSA). The ratio of Pt and Au contents on a single magnetic bead could be directly used for revealing the intrinsic proportion of fPSA in tPSA. In addition, good selectivity is shown in Fig. S14,† ensuring the specificity of the SMB nanoplatform.

### Standard-free prostate disease differentiation

Massive tPSA screening has been regarded as a candidate marker for monitoring prostate disease. Nevertheless, the use of solo tPSA measurement is greatly hampered by the fact that elevated serum tPSA concentration is also found in benign prostate conditions due to the heterogeneity between molecular subtypes.^41^ In order to provide a standardized nanoplatform for distinguishing benign prostate disease and prostate cancer, a 134-patient model containing 46 healthy donors, 44 BPH, and 44 prostate cancer patients was evaluated. We utilized the SMB nanoplatform for simultaneous evaluation of fPSA and tPSA and applied the Pt/Au ratio for depicting the biological proportion between fPSA and tPSA.

**Fig. 5:**
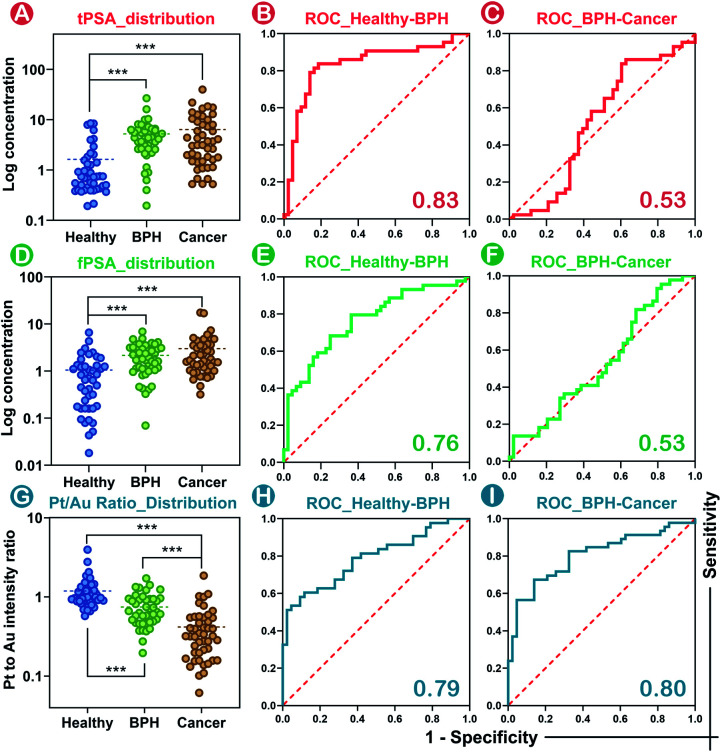
Distributions of solo tPSA (A), solo fPSA (D) and the Pt/Au ratio (G) among healthy donors, BPH and prostate cancer patients (Dotted line represents the mean value of the expression. *p < 0.05, **p < 0.01, ***p < 0.001). The distinction between BPH and cancer using solo tPSA or fPSA was tiny (*p* was 0.67 for solo tPSA and 0.59 for solo fPSA). (B) and (C) illustrate the ROC curves of solo tPSA for differentiation between healthy and BPH, and BPH and cancer. (E) and (F) illustrate the ROC curves of solo fPSA for differentiation between healthy and BPH, and BPH and cancer. (H) and (I) illustrate the ROC curves of the Pt/Au ratio for differentiation between healthy and BPH, and BPH and cancer.

Consistent with the conclusions of previous literature studies,^41,42^ higher levels of tPSA, fPSA, and lower percentages of fPSA (Pt/Au ratio) were found in patients with worse prostate conditions (Fig. 5A, D and G). In prostate disease differentiation, it was found that the Pt/Au ratio (equivalent to fPSA%) showed more significant differences among healthy, BPH, and cancer donors (Fig. 5G) compared with solo tPSA or fPSA (Fig. 5A and D, *P* was 0.67 for solo tPSA and 0.59 for solo fPSA). Receiver operating characteristic (ROC) analysis revealed that the Pt/Au ratio considerably differentiated healthy donors from unhealthy individuals (healthy *vs.* BPH) with an area under the curve (AUC) of 0.79 (shown in Fig. 5H), and exhibited improved differentiation accuracy of cancer from BPH in the unhealthy group (BPH *vs.* cancer, AUC was 0.80 in Fig. 5I). In contrast, the difference between BPH and cancer by using single tPSA or fPSA was tiny (Fig. 5A and D), and the AUC was only 0.53 using either solo tPSA or fPSA for differentiation (Fig. 5C, F, and S15†). Thresholds of the Pt/Au ratio for the differentiation of different groups were calculated and are provided in Table 1. Based on our model, a Pt/Au ratio of 0.765 could be considered for separating healthy or unhealthy samples, while a Pt/Au ratio of 0.434 could be applied for BPH-cancer differentiation in unhealthy individuals. By providing stable outcomes, the proposed SMB nanoplatform showed great potential in establishing universal and uniform criteria for prostate disease differentiation without the need for reference standards and will serve as a synergistic tool in cancer diagnosis, staging, and postoperative assessments in the future.

**Table 1:** Thresholds in prostate disease differentiation using the Pt/Au ratio

Differentiation	AUC	*p* value	Threshold	Sensitivity	Specificity
Healthy *vs.* unhealthy	0.79	<0.001	0.765	88.4%	60.5%
BHP *vs.* cancer	0.80	<0.001	0.434	86.0%	67.4%

## Conclusion

In summary, we proposed a single magnetic bead (SMB) nanoplatform for sensitive yet stable bioassays and demonstrated its standard-free analytical performance towards simultaneous evaluations of two key prostate cancer-related biomarkers (fPSA and tPSA) and the discovery of biological proportion within the PSA subtypes for prostate disease differentiation. The stable SMB platform has been realized by judiciously addressing key challenges associated with the size of metal nanoparticle probes in sphere magnetic bead-centered immunoassay. The inaccuracy from fluctuating total probe concentrations was also successfully diminished *via* evaluating the Au or Pt intensities on the single magnetic bead. Under optimal conditions, the SMB nanoplatform exhibited wide linear ranges as well as low LODs for both tPSA and fPSA evaluations, demonstrating the feasibility of standard-free analysis. In prostate disease differentiation, the use of the Pt/Au intensity ratio showed considerable differentiation towards healthy and unhealthy, BPH and cancer individuals, surpassing the use of solo tPSA or fPSA for differentiation between BPH and cancer patients. We generated thresholds for differentiating healthy and unhealthy, BPH and cancer patients, hoping to provide suggestions for cancer differentiation. By directly evaluating the proportion between a biomarker and its subtypes, the proposed SMB nanoplatform opens a new realm in standardized and stable data acquisition and processing platform constructions and can provide uniform criteria for users across the globe for efficient diagnosis of malignancy without biased decisions. Our ongoing work is to expand the application of the SMB platform in multiplex nucleic acid quantifications, proteinase screening, and immune response efficiency studies, and establish standard-free universal nanoplatforms for other malignancies worldwide.

## Data availability

The datasets supporting this article have been uploaded as part of the ESI.† See https://doi.org/10.1039/d2sc00928e.

## Author contributions

Zili Huang and Rui Liu conceptualized the project methodology. Zili Huang designed and implemented the experiments, collected and analyzed the experimental data, and wrote the original manuscript. Bei Xu provided all the serum samples from Mianyang Central Hospital. Zili Huang, Jianyu Hu, Xiaobo Xie, and Rui Liu revised the manuscript. Rui Liu and Yi Lv supervised the investigation.

## Conflicts of interest

There are no conflicts to declare.

## Supplementary Material

SC-013-D2SC00928E-s001
