# Supplementary material for: Standard-free single magnetic bead evaluation: a stable nanoplatform for prostate disease differentiation
Source: Chem Sci. 2022 Apr 29;13(21):6270–5. doi: 10.1039/d2sc00928e (PMC9159090; doi:10.1039/d2sc00928e)
Supplement: SC-013-D2SC00928E-s001 [file SC-013-D2SC00928E-s001.pdf]

Electronic Supplementary Material (ESI) for Chemical Science.
This journal is © The Royal Society of Chemistry 2022

Supporting Information

Standard-Free Single Magnetic Bead Evaluation: A Stable Nanoplatform for Prostate Disease Differentiation

Zili Huang, [a] Xiaobo Xie, [b] Bei Xu, [c] Rui Liu [a], Jianyu Hu [d] and Yi Lv [a,b*]

[a] Key Laboratory of Green Chemistry & Technology, Ministry of Education, College of Chemistry, Sichuan University, Chengdu, Sichuan 610064, China

[b] Analytical & Testing Center, Sichuan University, Chengdu 610064, China

[c] Department of Clinical Laboratory, Mianyang Central Hospital, School of Medicine, University of Electronic Science and Technology of China, Mianyang, China

[d] Division of Analytical and Environmental Toxicology, Department of Laboratory Medicine and Pathology, Faculty of Medicine & Dentistry, University of Alberta, Edmonton, Alberta T6G 2G3, Canada

*Corresponding Author

Yi Lv, E-mail: lvy@scu.edu.cn. Tel. and Fax: +86-28-8541-2798.

Table of Contents

Table of Contents 2
Experimental Procedures 3
Material 3
Apparatus 3
Synthesis and Labeling of Nanoparticle Probes 3
Establishment of SMB Nanoplatform 3
Simultaneous Evaluation of tPSA and fPSA 4
Selectivity and Stability 4
Statistical Analysis 4
Results and Discussion 5
Table S1. Working Conditions of ICPMS Based SMB Nanoplatform 5
Table S2. Recovery of SMB Nanoplatform in Serological Evaluation 5
Table S3. Nanoparticle Concentrations Measurements 6
Table S4. Comparison of Dual-Biomarkers Immunoassay for fPSA and tPSA analysis 6
Fig. S1: Transient $^{58}Fe^{+}$ Signals of Magnetic Beads in sp-ICP-MS 8
Fig. S2: Identification and Differentiation of AuNPs and PtNPs by Appearances and TEM Elemental Mapping 9
Fig. S3: Characterizations of PtNPs with Three Different Diameters 10
Fig. S4: Characterizations of AuNPs with Three Different Diameters 11
Fig. S5: UV Spectrometry of AuNPs and PtNPs 12
Fig. S6: Concentration Measurements of AuNPs and PtNPs 13
Fig. S7: Relationship Between Nanoparticle Diameter and Metal Isotopic Intensity in sp-ICP-MS 14
Fig. S8: Labeling of AuNPs and PtNPs 15
Fig. S9: Optimization on Volume of Magnetic Beads in Immunoassay 16
Fig. S10: Optimization on Wash Times of Magnetic Beads in Immunoassay 17
Fig. S11: Transient $^{194}Pt^{+}$ Signals on Magnetic Beads During Immunoassay 18
Fig. S12: Transient $^{197}Au^{+}$ Signals on Magnetic Beads During Immunoassay 19
Fig. S13: Frequency distribution of Pt/Au content on Single Magnetic Bead 20
Fig. S14: Selectivity of the SMB Nanoplatform 21
Fig. S15: ROC Curves for Prostate Disease Differentiation 22
References 23

Experimental Procedures

Material

Chloroplatinic acid hydrate ($H_2PtCl_6 \cdot xH_2O$), chloroauric acid hydrate ($HAuCl_4 \cdot xH_2O$), potassium borohydride (KBH_4), 3-(3-dimethyl aminopropyl)-1-ethyl carbodiimide hydrochloride (EDC), N-hydroxysulfosuccinimide sodium (sulfo-NHS), sodium tetraborate, orthoboric acid, sodium phosphate-buffered saline (PBS), and L(+)-ascorbic acid were purchased from Adamas Reagent, Ltd. (Shanghai, China). 4-morpholineethanesulfonic acid (MES) was from Aladdin Chemistry Co. Ltd. Prostate disease-related biomarkers containing coating-anti-tPSA/fPSA antibodies, reporting-anti-tPSA/fPSA antibodies, tPSA, and fPSA antigens were purchased from Shanghai Linc-Bio Science Co. Ltd. (Shanghai, China). Bovine serum albumin (BSA) was from Shanghai Sangon Biotechnology Co. Ltd. (Shanghai, China). Commercial Dynabeads™ (MyOne™) Carboxylic Acid magnetic beads were purchased from Thermo Fisher Scientific Inc. Ultrapure water with 18.24 MΩ cm^{-1} came from a UPURE Sichuan water purification system. All serum samples were obtained from Mianyang Central Hospital. Hematoxylin-eosin (H&E) staining and immunohistochemical assay were implemented for prostate disease confirmation. The study was approved by the Medical Ethics Committee of Mianyang Central Hospital. Part of serum samples with known fPSA and tPSA concentrations were measured by ARCHITECT *i2000* from Abbott.

Apparatus

Absorption of AuNPs and PtNPs with three different diameters were measured by Cytation 5 Cell Imaging Multi-Mode Reader (BioTek Instruments, Inc.) using polystyrene 96-well microtiter plates (Jet Bio-Filtration Co., Ltd.). Diameters of nanoparticles were implemented by Malvern Nano ZS90 (Malvern Panalytical Ltd.). NexION-350 inductively coupled plasma mass spectrometer (PerkinElmer, Inc.) with single particle mode was applied for data collecting in SMB nanoplatform. Working conditions of SMB nanoplatform were listed in Table S1. Transmission electron microscopy (TEM) images were obtained by a JEM-2010 microscope (JEOL Co., Japan) and diameters of nanoparticles were calculated using ImageJ (1.52a version).

Synthesis and Labeling of Nanoparticle Probes

A series of AuNPs with different diameters were synthesized according to former articles with slight modifications.[1, 2] Briefly, 1.1 mL 1% trisodium citrate was added in a three-necked flask with 75 mL ultra-pure water. After boiling for 10 min, $HAuCl_4$ (0.01%, w/v) was added and kept refluxing for 20 min. Then the solution was cool down to 90 ^oC, followed by adding 0.5 mL $HAuCl_4$ (0.01%, w/v) into the solution again. The procedure was repeated one more time to obtain 21 nm AuNPs. 31 nm AuNPs were synthesized by repeating the above procedure three times. 47 nm AuNPs were synthesized by one-step reduction where 1 mL 1% trisodium citrate was added to boiling solution containing 100 mL ultrapure water and 0.01% $HAuCl_4$.

The synthesis of PtNPs with three different diameters was carried out based on the seed growth method.[3] Firstly, 5 nm Pt seeds were synthesized by successively adding a mixture of 1% trisodium citrate and 0.05% citrate acid, 0.55 mL 1% trisodium citrate, and 0.1% KBH_4 to 50 mL boiling solution containing 0.2% chloroplatinic acid. Secondly, 1% chloroplatinic acid and 1.25% ascorbic acid was mixed with 2 mL 5 nm Pt seeds and ultrapure water in a three-necked flask, followed by boiling and refluxing to obtain 18 nm PtNPs. 36 nm and 51 nm PtNPs were synthesized respectively by adding 11 mL and 8 mL 18 nm PtNPs in a three-necked flask together with ultrapure water, 1% chloroplatinic acid, and trisodium citrate/ascorbic acid mixture. All as-synthesized nanoparticles were purified by centrifuge and characterized by UV, TEM, DLS, and sp-ICP-MS.

Establishment of SMB Nanoplatform

Magnetic bead-centered SMB nanoplatform was established by preparing antibody labeled magnetic beads. Briefly, 20 μL 10mg/mL carboxylic acid-coated magnetic beads were firstly activated by EDC (150 mg/mL) and sulfo-NHS (25 mg/mL) under the 6.5 pH condition. MES solved coating-anti-tPSA antibody was then mixed with magnetic beads. Coupling between carboxyl on magnetic beads and amino groups on antibodies took 12 hours. Antibody labeled magnetic beads were finally blocked by 10% BSA-contained PBS buffer and washed three times by 1% BSA-contained PBS buffer.

Concentrations of both AuNPs and PtNPs with three different diameters were counted by the digestion method mentioned in our previous work.[4] Nanoparticles were digested by aqua regia ($1HNO_3$:3HCl, v/v) and ions concentrations were subsequently measured by standard mode (Shown in Fig. S6).

After adjusting the nanoparticle concentrations to an appropriate range, nanoparticle probes were synthesized by electrostatic attachments. Primarily, 1 mL boric acid buffer dispersed AuNPs (pH 8.2) were mixed with 30 μL 1 mg/mL labeling-anti-tPSA antibody which was optimal as the best volume according to our previous work.[4] The attachment took 1 hour, followed by another surface blocking hour by 10% BSA PBS buffer. Antibody labeled AuNPs were purified from excessive antibody and BSA by 16 min centrifugation. 500 μL 1% BSA PBS buffer was applied to redisperse the nanoparticles after discarding the supernatant. The scenario of PtNPs labeling was carried in the similar way. Mixed probes were synthesized by adding as-prepared 500 μL AuNPs probes and 500 μL PtNPs probes into a 1.5 mL centrifugal tube.

Simultaneous Evaluation of tPSA and fPSA

Under the fact that fPSA is a subtype of tPSA which is not bound with protease inhibitors,[5] standard curves of fPSA and tPSA were implemented separately in this work. The standard curve of fPSA was implemented by mixing varied concentrations of fPSA with mixed probes, while the standard curve of tPSA was carried out by mixing tPSA with mixed probes. Simultaneous detection of tPSA and fPSA in serum samples by SMB nanoplatform was implemented as followed: 5 μg antibody labeled magnetic beads were added in a 200 μL PCR tube, followed by adding 60 μL diluted serum samples and 25 μL mixed probes. All serum sample was diluted at least two times to reduce matrix effects. Samples were incubated under 37 ^{o}C for 2 hours, followed by two times washing using 1% BSA PBS buffer containing 0.05-0.1% Tween-20. Before SMB detection, samples were diluted 400 times using ultrapure water. In sp-ICP-MS mode, $^{58}Fe^{+}$ was applied for magnetic beads counting, making all measurements under the same number of magnetic beads. $^{194}Pt^{+}$ and $^{197}Au^{+}$ were chosen for evaluating Pt and Au intensity on each magnetic bead.

Selectivity and Stability

Selectivity of the proposed SMB nanoplatform was carried out by mixing nanoparticle probes and magnetic beads with tPSA/fPSA (50 ng/mL for both) or interferences (500 ng/mL for IgG, 500 ng/mL for AFP, 500 ng/mL for CEA, 500 U/mL for CA199, 500 U/mL for CA125 and 500 U/mL for CA153). Measurements of Pt and Au were carried out simultaneously by sp-ICP-MS. The stability of the SMB nanoplatform was explored by successively applying immunoassay using 50 ng/mL fPSA and 50 ng/mL tPSA.

Statistical Analysis

In this article, the average intensity of Au or Pt per magnetic bead was calculated using the formula:

$$\bar{I} = \frac{\sum IN}{\sum N}$$

Where $\bar{I}$ was the average intensity of Au or Pt per magnetic bead, I represented the intensity of Au or Pt of single magnetic bead, N represented the number of magnetic beads.

Data processing was performed using Origin 2018. Statistical analysis of 134 serum samples was implemented by Prism 8.0.2. Comparisons of concentration between healthy donors, BPH and prostate cancer patients were carried performed by Kruskal-Wallis Test. Receiver operating characteristics (ROC) analysis was carried out using IBM SPSS statistics 26. tPSA data analysis was carried out using 43 healthy, 43 BPH, and 46 cancer individuals. fPSA data analysis was carried out using 44 healthy, 44 BPH, and 44 cancer individuals.

Results and Discussion

Table S1. Working Conditions of ICPMS Based SMB Nanoplatform

Parameters	Values
Au (*m/z*)	197 (100%)
Pt (*m/z*)	194 (33.0%)
Fe (*m/z*)	58 (0.3%)
ICP RF Power (W)	1300
Plasma Gas Flow (L/min)	18
Auxiliary Gas Flow (L/min)	1.20
Nebulizer Gas Flow (L/min)	0.93
Deflector Voltage (V)	11.75
Pulse Stage Voltage (V)	1200
Analog Stage Voltage (V)	1925
Sample Uptake Rate (mL/min)	0.25
Dwell Time (μs)	200
Detecting Time (s)	10

Table S2. Recovery of SMB Nanoplatform in Serological Evaluation

Type	Sample (ng/mL)	Add (ng/mL)	Found (ng/mL)	Recovery (%)
fPSA	1.78	--	1.85	--
		5	4.26	85
		10	11.8	118
		20	22.9	114
	4.48	--	4.51	--
		5	4.27	85
		10	8.82	88
		20	17.8	89
	0.097	--	0.086	--
		5	5.12	102
		10	9.82	98
		20	23.0	115
tPSA	0.71	--	0.76	--
		5	4.59	92
		10	9.56	96
		20	18.1	91
	9.11	--	9.44	--
		5	5.52	110
		10	11.9	119
		20	23.5	117
	11.4	--	12.0	--
		5	4.79	96
		10	9.15	92
		20	18.7	94

Table S3. Nanoparticle Concentrations Measurements

External standard method was applied for measuring the ion concentrations of AuNPs and PtNPs after digestion by aqua regia. Concentrations of nanoparticles were calculated using formula (external standard linears were shown in Fig. S6, *Concentration* was number concentration of nanoparticles, C_{Ions} was the ion concentration calculated by external standard of Au of Pt after digestion by aqua regia. D represented the diameter of nanoparticle. N_A represented the Avogadro constant.):

$$Concentration = \frac{6C_{Ions}}{\pi\rho D^3 N_A}$$

Nanoparticles	Diameter / nm	Concentration / pmol·L^{-1}	Average / pmol·L^{-1}	SD
AuNPs	21±3	361	360	1.66
		360		
		358		
	31±3	132	130	3.04
		132		
		127		
	47±3	95	100	4.27
		101		
		103		
PtNPs	18±2	305	305	0.42
		304		
		305		
	36±2	258	260	4.10
		259		
		265		
	51±3	105	105	0.31
		104		
		105		

Table S4. Comparison of Dual-Biomarkers Immunoassay for fPSA and tPSA analysis

Biomarkers	Method	Linear range	LOD	refs
tPSA/fPSA	Time-resolved fluoroimmunoassay	tPSA: 0.5-50 ng/mL<br>fPSA: 0.5-50 ng/mL	tPSA: 0.1 ng/mL<br>fPSA: 0.01 ng/mL	6
tPSA/fPSA	Fluorescence resonance energy transfer (FRET)	tPSA: 0.60-100 ng/mL<br>fPSA: 0.74-100 ng/mL	--	7
cPSA/fPSA	Surface-enhanced Raman scattering (SERS)	fPSA: 0.005-50 ng/mL<br>cPSA: 0.045-450 ng/mL	fPSA: 0.012 ng/mL<br>cPSA: 0.15 ng/mL	8
cPSA/fPSA	MQBs-based fluorescent lateral flow test strip	fPSA: 0.05-20 ng/mL<br>cPSA: 0.2-80 ng/mL	fPSA: 0.009 ng/mL<br>cPSA: 0.087 ng/mL	9
tPSA/fPSA	Screen-printed electrochemical dual sensors	fPSA: 1-10 ng/mL<br>tPSA: 1-10 ng/mL	fPSA: 1.0 ng/mL<br>tPSA: 1.0 ng/mL	10
tPSA/fPSA	SERS-based microfluidic device	tPSA: 0.05-100 ng/mL<br>fPSA: 0.005-10 ng/mL	tPSA: <0.1 ng/mL<br>fPSA:<0.1 ng/mL	11
tPSA/fPSA	Surface plasmon resonance immunoassay	tPSA: 0.085-0.25 ng/mL<br>fPSA: 0.01-0.4 ng/mL	tPSA: 0.03 ng/mL<br>fPSA: 0.005 ng/mL	12
tPSA/fPSA	Chemiluminescence immunoassay	tPSA: 0.02-125 ng/mL<br>fPSA: 0.01-36.7 ng/mL	tPSA: 0.007 ng/mL<br>fPSA: 0.004 ng/mL	13
tPSA/fPSA	Chemiluminescence enzyme immunoassay	--	tPSA: 0.05 ng/mL<br>fPSA: 0.03 ng/mL	14

tPSA/fPSA	Rapid bead-based immunoassay	tPSA: 0.8-156.6 ng/mL<br>fPSA: 0.1-18.5 ng/mL	tPSA: 0.0023 ng/mL<br>fPSA: 0.0013 ng/mL	15
tPSA/fPSA	DELFIA prostatus dual assay (commercial)	--	tPSA: 0.05 ng/mL<br>fPSA: 0.04 ng/mL	16
tPSA/fPSA	Time-resolved fluoroimmunoassay	tPSA: 0.5-100 ng/mL<br>fPSA: 0.5-100 ng/mL	tPSA: 0.05 ng/mL<br>fPSA: 0.006 ng/mL	17
tPSA/fPSA	Optical trapping assisted bead-array based fluorescent assay	tPSA: 0.01-40 ng/mL<br>fPSA: 0.01-10 ng/mL	tPSA: 0.0025 ng/mL<br>fPSA: 0.0038 ng/mL	18
cPSA/fPSA	Microcantilevers	fPSA: 0.2-60000 ng/mL<br>cPSA: 0.2-60000 ng/mL	--	19
tPSA/fPSA	Immunoluminometric sandwich assay (LIAISON, commercial)	tPSA:0.09-300 ng/mL<br>fPSA:0.04-25 nm/mL	tPSA: 0.04 ng/mL<br>fPSA: 0.09 ng/mL	20
tPSA/fPSA	Time resolved amplified cryptate emission technology (BRAHMS Kryptor, commercial)	tPSA: 0-70 ng/mL<br>fPSA: 0-15 ng/mL	tPSA: 0.04 ng/mL<br>fPSA: 0.02 ng/mL	
tPSA/fPSA	Chemiluminescent Microparticle (Architect, commercial)	tPSA: 0-100 ng/mL<br>fPSA: 0-30 ng/mL	tPSA: <0.008 ng/mL<br>fPSA: <0.008 ng/mL	
tPSA/fPSA	Chemiluminescent paramagnetic Particles (Access Hybritech, commercial)	tPSA: 0.008-150 ng/mL<br>fPSA:0.005-20 ng/mL	tPSA: 0.008 ng/mL<br>fPSA: 0.005 ng/mL	
tPSA/fPSA	Microparticle Enzyme Immunoassay (AxSym, commercial)	tPSA: 0.04-50 ng/mL<br>fPSA: 0.02-10 ng/mL	tPSA: 0.06 ng/mL<br>fPSA: 0.02 ng/mL	
cPSA/fPSA	Chemiluminescence (ADVIA Centaur XP/CP, commercial)	cPSA: 0.03-100 ng/mL<br>fPSA: 0.01-25 ng/mL	cPSA: 0.03 ng/mL<br>fPSA: 0.01 ng/mL	
tPSA/fPSA	Chemiluminescence (Immulite, commercial)	tPSA: 0.04-150 ng/mL<br>fPSA: 0.07-25 ng/mL	-	
tPSA/fPSA	Chemiluminescence (Dimension RxL, commercial)	tPSA: 0.13-100 ng/mL<br>fPSA: 0.06-45 ng/mL	tPSA: 0.13 ng/mL<br>fPSA: 0.06 ng/mL	
tPSA/fPSA	Chemiluminescence (Dimension Vista, commercial)	tPSA: 0.01-100 ng/mL<br>fPSA: 0.015-20 ng/mL	tPSA: 0.008 ng/mL<br>fPSA: 0.005 ng/mL	
tPSA/fPSA	Electrochemiluminescence (Elecsys, commercial)	tPSA: 0.002-100 ng/mL<br>fPSA: 0.01-50 ng/mL	tPSA: 0.002 ng/mL<br>fPSA: 0.01 ng/mL	
tPSA/fPSA	Two step immune-sandwich assay (VIDAS, commercial)	tPSA: 0.07-100 ng/mL<br>fPSA: 0.05-10 ng/mL	tPSA: 0.07 ng/mL<br>fPSA: 0.05 ng/mL	
tPSA/fPSA	Biochip array technology (Evidence Biochip Analyser, commercial)	tPSA: 0-100 ng/mL<br>fPSA: 0-75 ng/mL	tPSA: 0.045 ng/mL<br>fPSA: 0.02 ng/mL	
tPSA/fPSA	Single magnetic bead (SMB) nanoplatform (this work)	tPSA: 0.05-100 ng/mL<br>fPSA: 0.05-100 ng/mL	tPSA: 0.021 ng/mL<br>fPSA: 0.012 ng/mL	-

Fig. S1: Transient $^{58}Fe^{+}$ Signals of Magnetic Beads in sp-ICP-MS

Magnetic beads counting was carried out using ^{58}Fe isotopes in sp-ICP-MS. Each pulse above the threshold (10 counts) represents a detected magnetic bead.

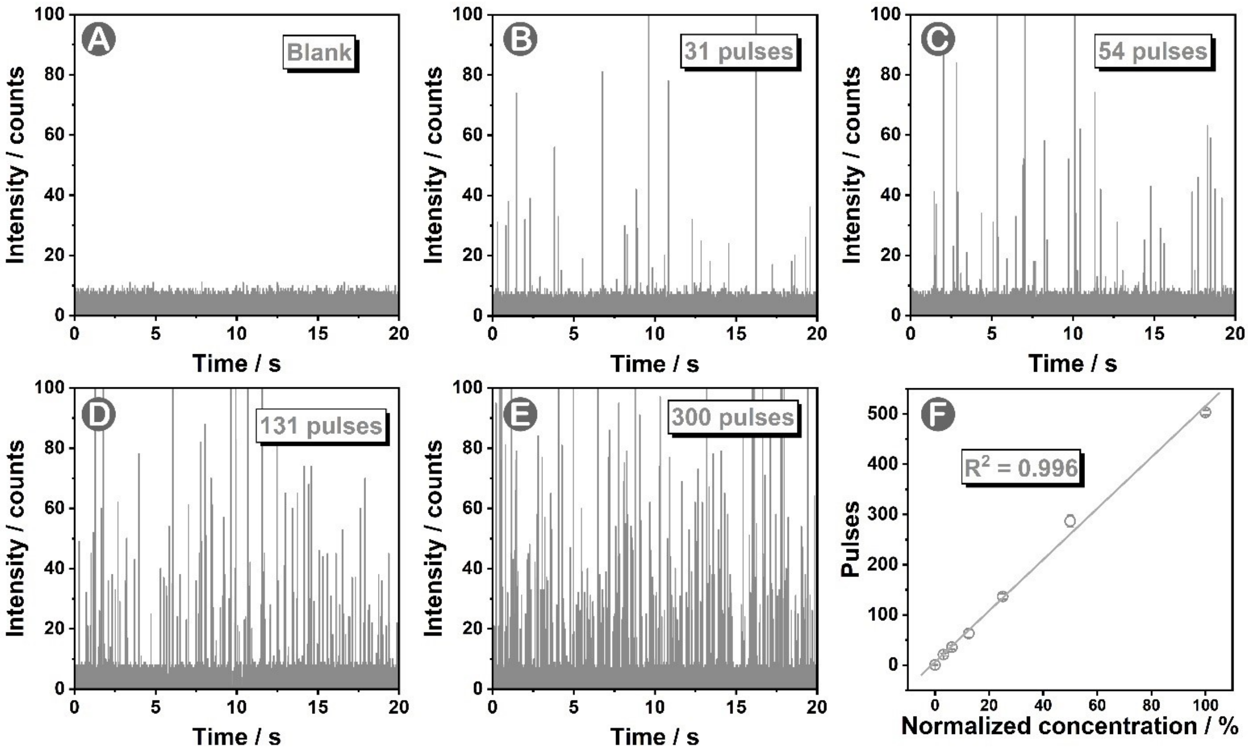

Fig. S1. Magnetic beads counting using $^{58}Fe^{+}$ isotopes. (a)-(e) illustrated the real-time signals of magnetic beads with increased concentration, while (f) showed the linear relationship between a number of magnetic beads and signals (pulses).

Fig. S2: Identification and Differentiation of AuNPs and PtNPs by Appearances and TEM Elemental Mapping

Due to the different appearances, AuNP and PtNPs were identified and differentiated by roughness on the surface (a). PtNPs were synthesized by seed growth method where crystallinity and roughness existed on the surface, while the surface of AuNPs was smooth. The captured AuNPs and PtNPs on a single magnetic bead were also observed by TEM mapping (b).

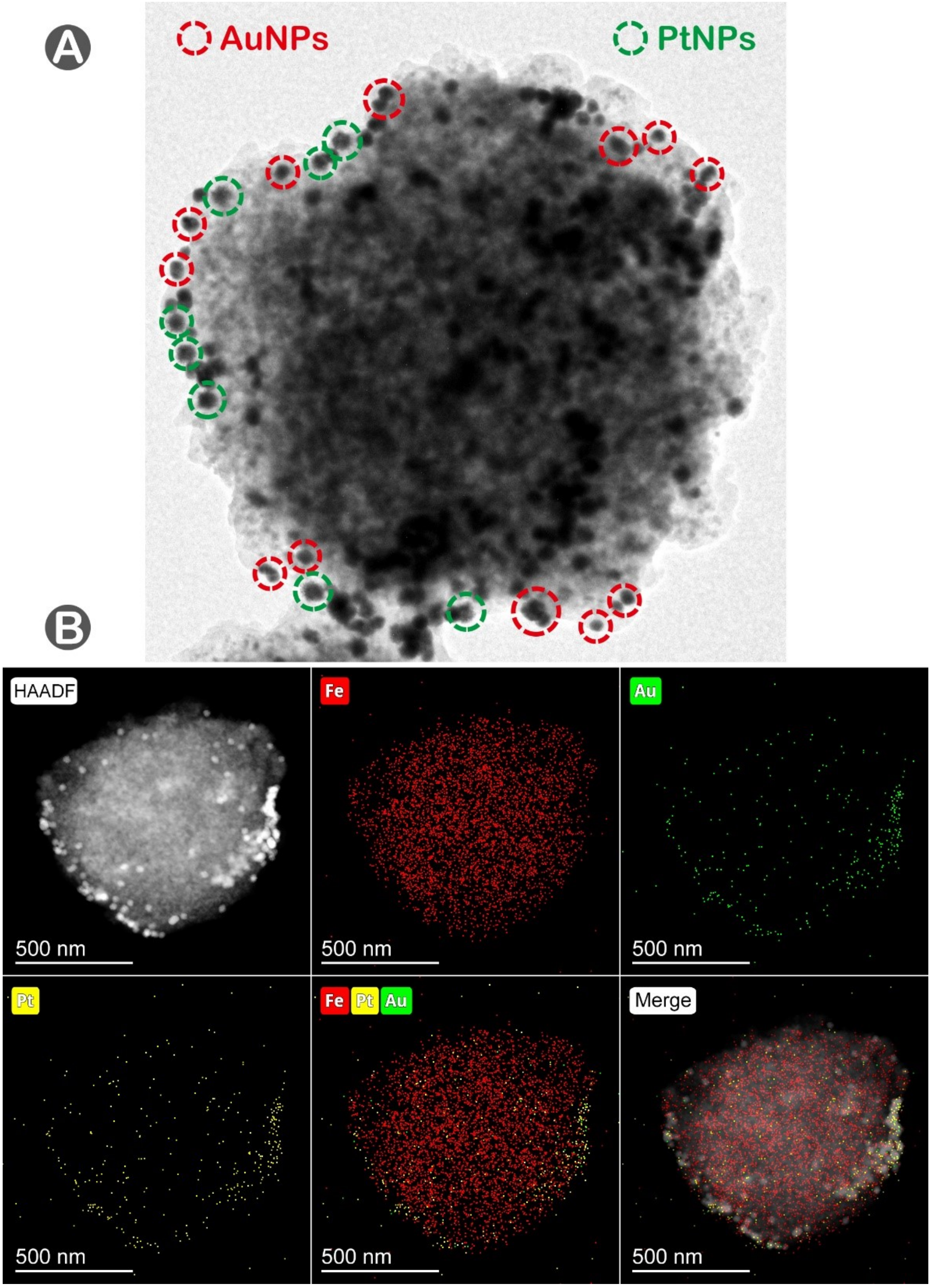

Fig. S2. Identification and differentiation of AuNPs and PtNPs on a single magnetic bead by nanoparticle appearances (a) and TEM mapping (b).

Fig. S3: Characterizations of PtNPs with Three Different Diameters

In this work, TEM was applied for getting the diameters of PtNPs. ImageJ (1.52a version) was used for the diameter analysis where the ruler in TEM image was considered as the standard. Generally, 18±2 nm, 36±2 nm and 51±3 nm PtNPs were synthesized for diameter-regulation immunoassays. DLS and sp-ICP-MS were also applied respectively for characterizations.

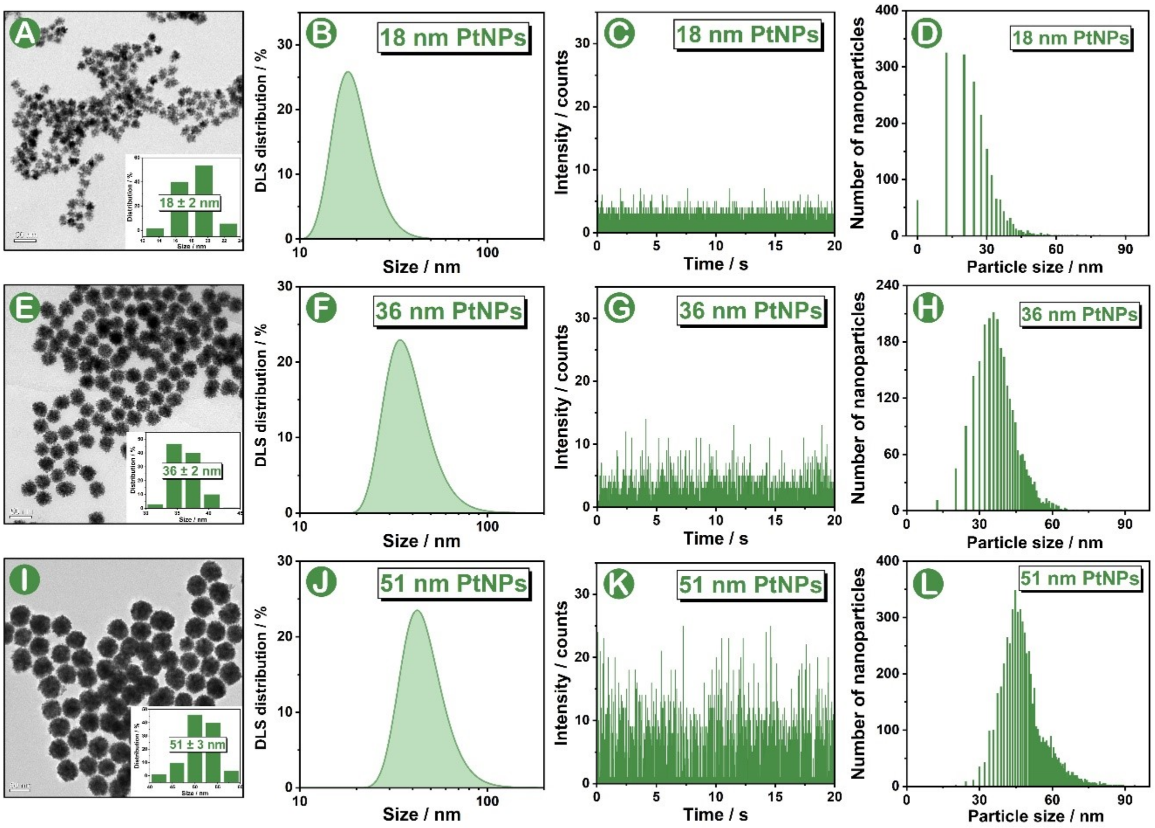

Fig. S3. Characterizations of as-prepared PtNPs. TEM (a), (e) and (i) were applied for diameter measuring. Inset images illustrated diameter analysis of nanoparticles using ImageJ. DLS (b), (f) and (j) were used for distribution analysis. Real-time signals (c), (g) and (k) of three different diameters were measured by sp-ICP-MS. The size distributions of PtNPs measured by sp-ICP-MS were exhibited in (d), (h) and (l) respectively.

Fig. S4: Characterizations of AuNPs with Three Different Diameters

In this work, TEM was applied for getting the diameters of AuNPs. ImageJ (1.52a version) was used for the diameter analysis where the ruler in TEM image was considered as the standard. Generally, 21±3 nm, 31±3 nm and 47±3 nm AuNPs were synthesized for diameter-regulation immunoassays. DLS and sp-ICP-MS were also applied respectively for characterizations.

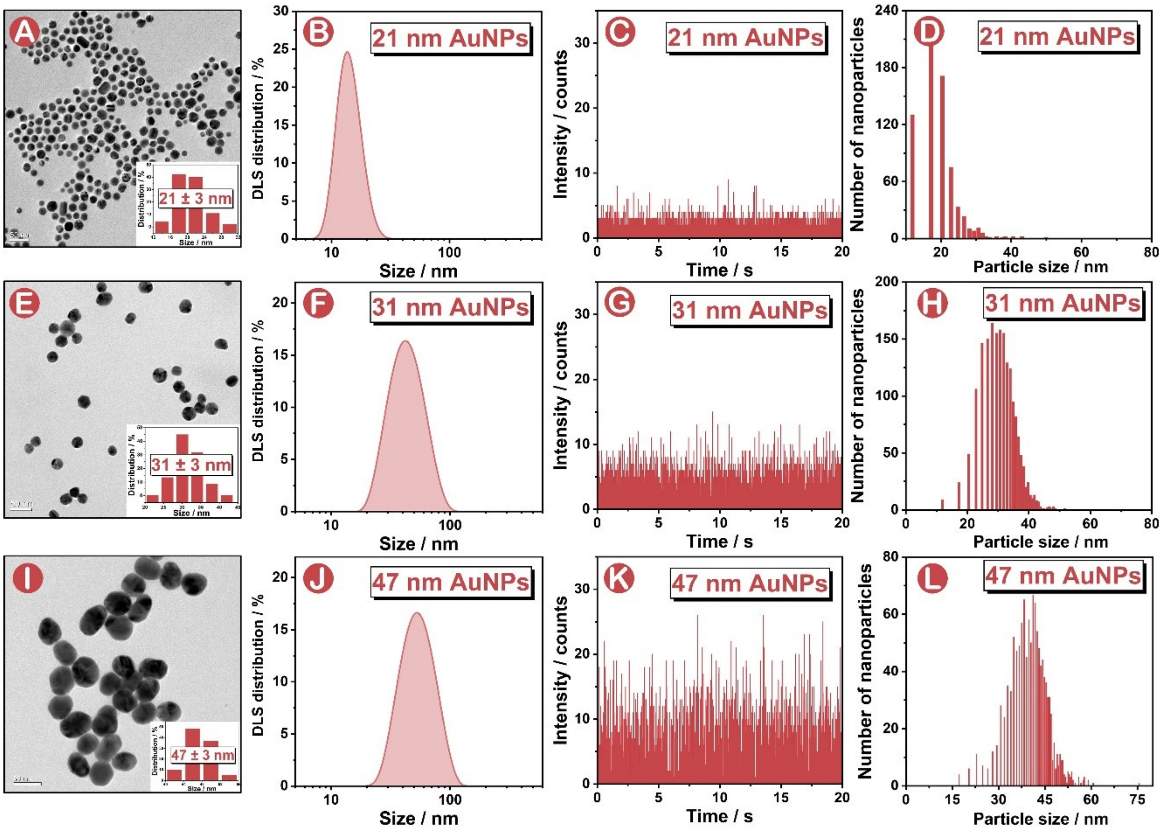

Fig. S4. Characterizations of as-prepared AuNPs. TEM (a), (e) and (i) were applied for diameter measuring. Inset images illustrated diameter analysis of nanoparticles using ImageJ. DLS (b), (f) and (j) were used for distribution analysis. Real-time signals (c), (g) and (k) of three different diameters were measured by sp-ICP-MS. The size distributions of AuNPs measured by sp-ICP-MS were exhibited in (d), (h) and (l) respectively.

Fig. S5: UV Spectrometry of AuNPs and PtNPs

UV was utilized to depict the peak of absorbance. The peaks of AuNPs were found as 520 nm for 21 nm, 528 nm for 31 nm, and 534 nm for 47 nm. The peaks of PtNPs were found as 237 nm for 18 nm, 251 nm for 36 nm, and 306 nm for 51 nm.

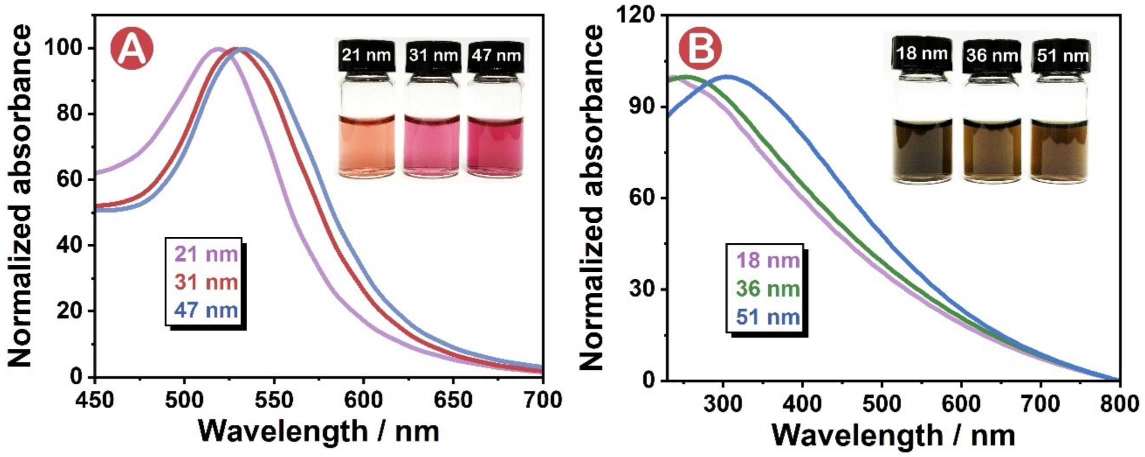

Fig. S5. UV spectrometry of AuNPs (a) and PtNPs (b).

Fig. S6: Concentration Measurements of AuNPs and PtNPs

External standard method was applied for measuring the ion concentrations of AuNPs and PtNPs after digestion by aqua regia. Concentrations of nanoparticles were calculated using formula (detailed nanoparticle concentrations were shown in Table S3):

$$C_{Nano}(number) = \frac{6C_{Ions}}{\pi \rho D^3 N_A}$$

Where C_{Nano} was number concentration of nanoparticles, C_{Ions} was ion concentration of Au of Pt after digestion by aqua regia. D represented the diameter of nanoparticle. N_A represented the Avogadro constant.

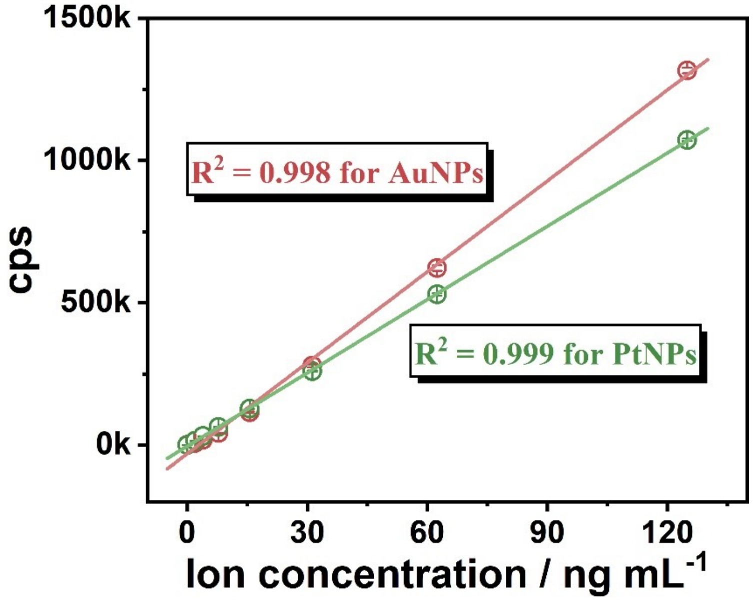

Fig. S6. Standard curves of between ion concentrations of nanoparticles and standard mode signals.

Fig. S7: Relationship Between Nanoparticle Diameter and Metal Isotopic Intensity in sp-ICP-MS

According to previous reports,[21] the relationship between the number of total atoms in each nanoparticle can be calculated as Formula a where N represents the number of atoms in each nanoparticle, while ρ is the metal density, M_{metal} is atomic weight and D represents the diameter. In sp-ICP-MS, the number of atoms is proportional to intensity (I) which is illustrated in Formula b, thus the relationship between diameter (d) and intensity can be calculated as Formula c (K is a constant) where intensity is proportional to d^3.

$$N = \frac{\pi \rho D^3}{6M_{metal}} \text{ (a)}$$

$$I = K_1 \times N \text{ (b)}$$

$$I = K_2 \times d^3 \text{ (c)}$$

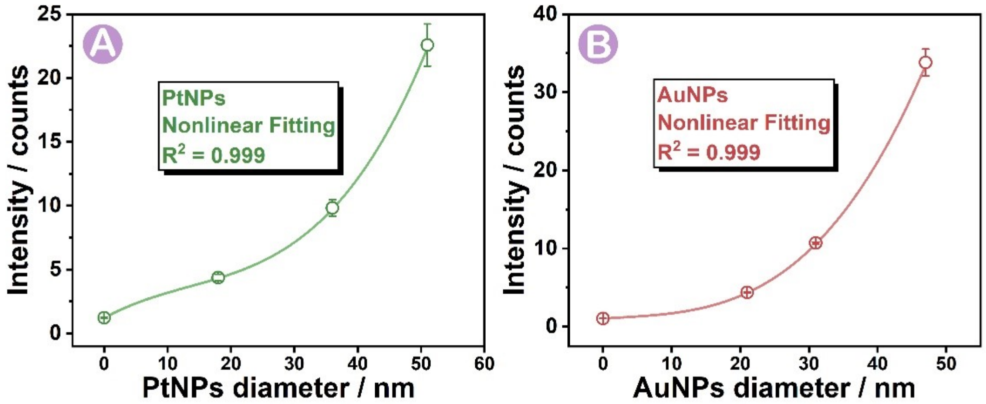

Fig. S7. Relationship Between Nanoparticle Diameter and Metal Isotopic Intensity in sp-ICP-MS.

Fig. S8: Labeling of AuNPs and PtNPs

DLS was applied for characterization of nanoparticles labeling. In accordance with the results, distribution of nanoparticles moved to bigger diameters, indicating that antibodies were loaded on the surface of nanoparticles. In addition, no changes in intensity of nanoparticles after labeling in sp-ICP-MS measurements, further proving the successful labeling.

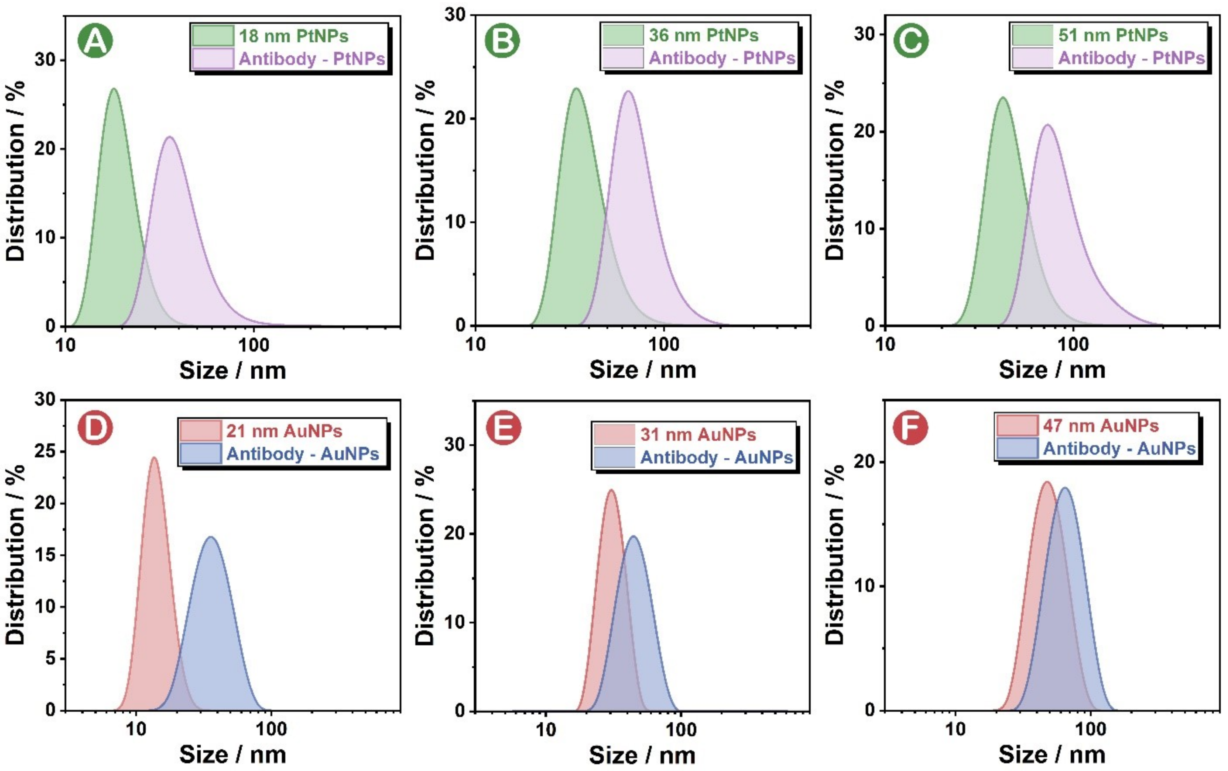

Fig. S8. Characterization of nanoparticles labeling using DLS. (a), (b) and (c) shown the distribution changes of AuNPs with different diameters. (d), (e) and (f) illustrated the distribution changes of PtNPs.

Fig. S9: Optimization on Volume of Magnetic Beads in Immunoassay

Optimization on volume of magnetic beads was carried using fPSA-PtNPs immunoassay. As shown below, both Pt and Fe signals increased when using bigger volume of magnetic beads. By evaluating the signal-to-noise ratios, the highest S/N signals were found using 5 μL magnetic beads. Lower volume of magnetic beads was also considered in optimizations but the separation in sample became difficult in return. Thus, we finally chose 5 μL magnetic beads in each sample (about 5 μg magnetic beads per sample).

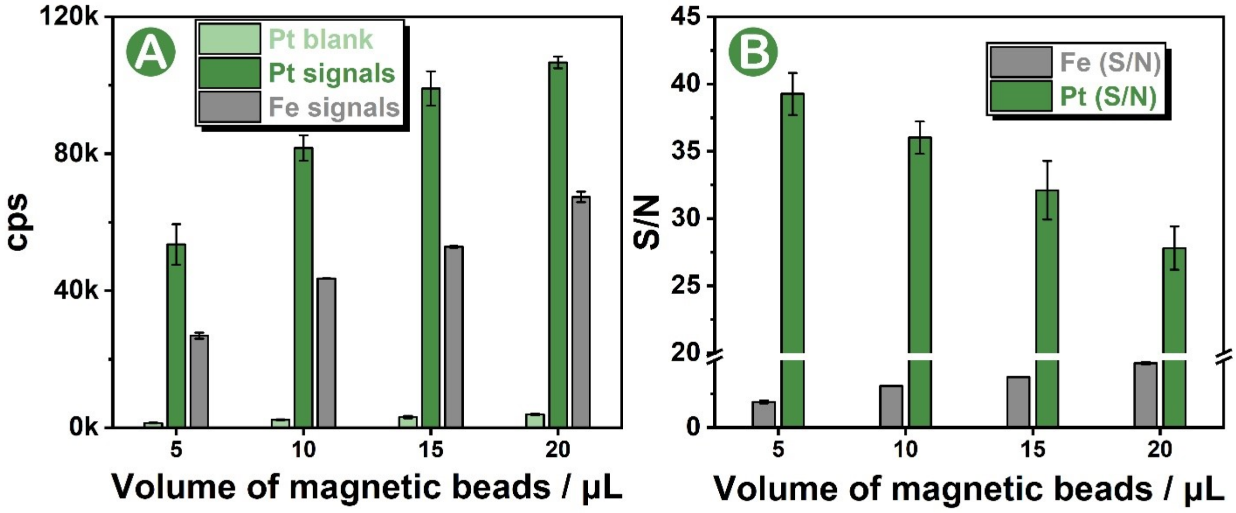

Fig. S9. Optimization on volume of magnetic beads in immunoassay. (a) signals of Pt, Pt blank and Fe. (b) S/N signals in optimization.

Fig. S10: Optimization on Wash Times of Magnetic Beads in Immunoassay

Optimization on wash times of magnetic beads was also carried using fPSA-PtNPs immunoassay. According to the results, S/N signals reached the maxima when taking two times wash steps.

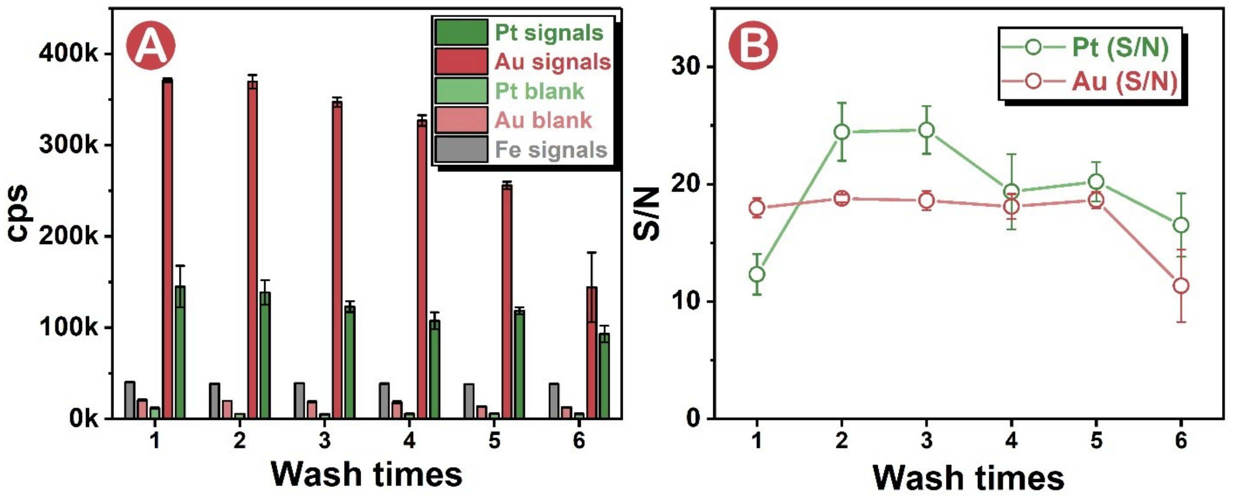

Fig. S10. Optimization on wash times of magnetic beads in immunoassay. (a) signals of Pt/Au, Pt/Au blank and Fe. (b) S/N signals of Au and Pt in optimization.

Fig. S11: Transient $^{194}Pt^{+}$ Signals on Magnetic Beads During Immunoassay

Single immunoassay of fPSA was carried using SMB nanoplatform. Under different concentration of fPSA, increased transient Pt signals were detected.

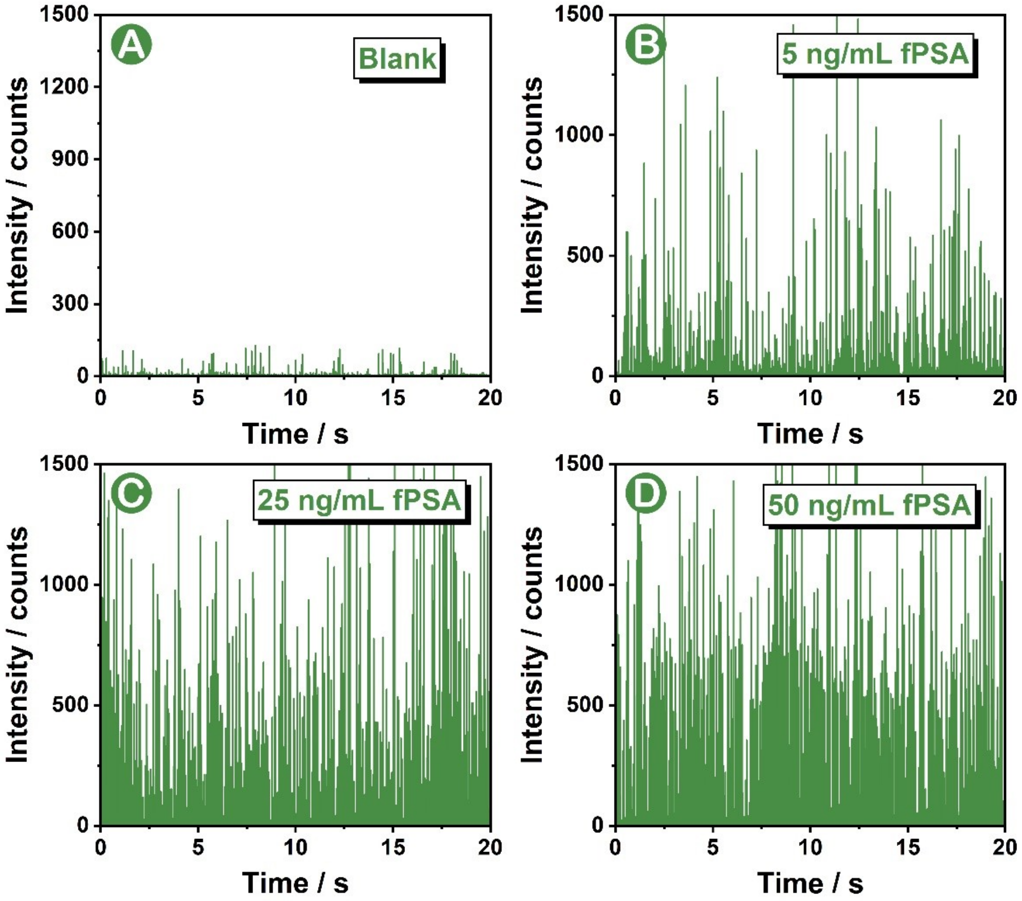

Fig. S11. sp-ICP-MS measurements of PtNPs probes which were spiked with different concentrations of fPSA.

Fig. S12: Transient $^{197}Au^{+}$ Signals on Magnetic Beads During Immunoassay

Single immunoassay of tPSA was carried using SMB nanoplatform. Under different concentration of fPSA, increased transient Au signals were detected.

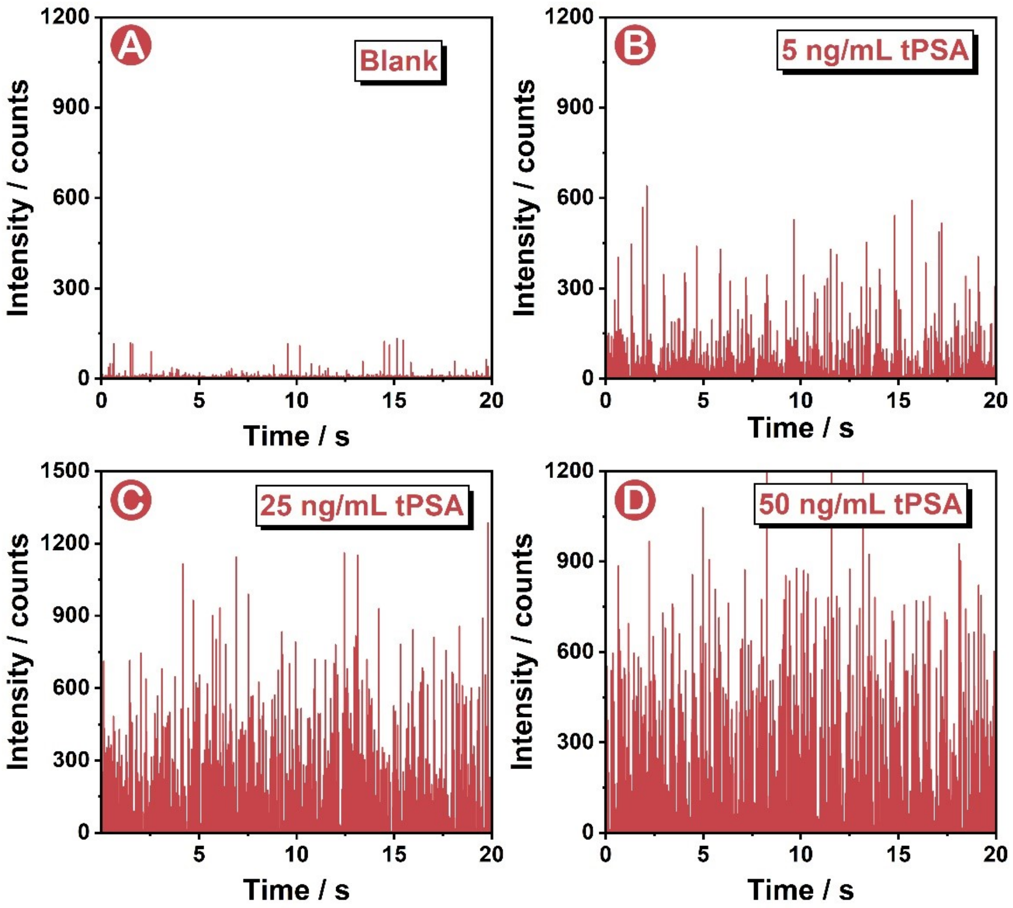

Fig. S12. sp-ICP-MS measurements of AuNPs probes which were spiked with different concentrations of tPSA.

Fig. S13: Frequency distribution of Pt/Au content on Single Magnetic Bead

After measuring magnetic beads by sp-ICP-MS under the different concentrations of relative antigens, frequency distributions were processed by applying the intensity of Au or Pt per magnetic bead as X coordinate, and the number of magnetic beads as Y coordinate. The background signals were subtracted to get the metal distributions. On the obtaining histograms, the first sample distribution (0 ng/mL) attributed to the signals of background and a small number of nanoparticles left in sample after two-steps washing.

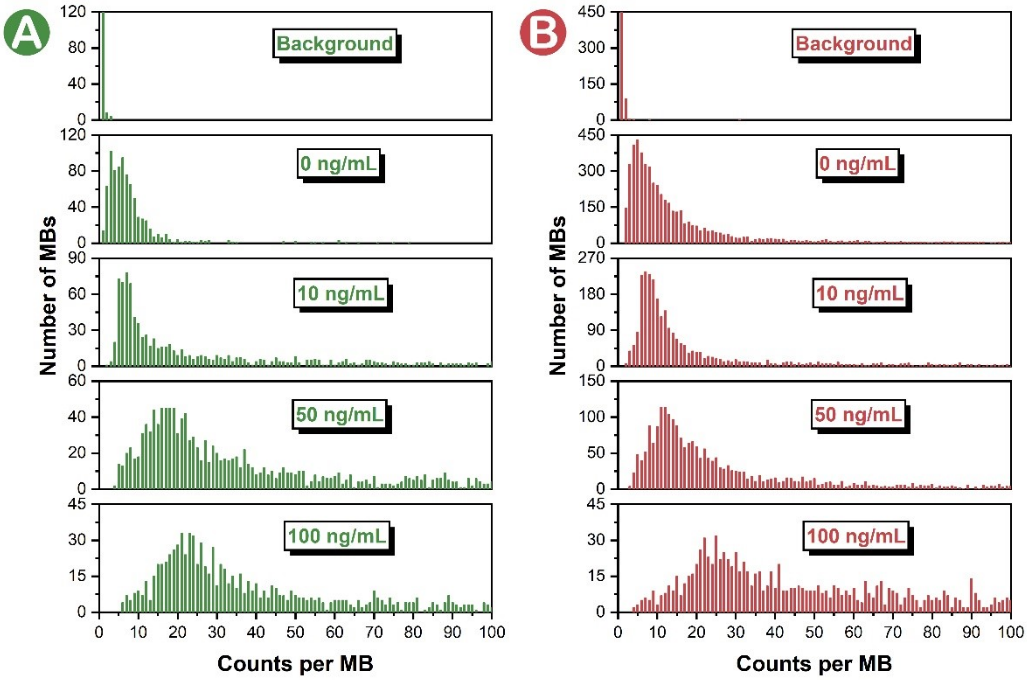

Fig. S13. Frequency distribution of metal content (a) for fPSA-PtNPs and (b) for tPSA-AuNPs on single magnetic bead. Background signals were got and subtracted in further detections by Perkin Elmer Syngistix^{TM} Software (1.1.4624.0 Version).

Fig. S14: Selectivity of the SMB Nanoplatform

Selectivity of the proposed SMB nanoplatform was carried by mixing nanoparticle probes and magnetic beads with tPSA/fPSA or interferences. Detail information was illustrated in Experimental Procedures part.

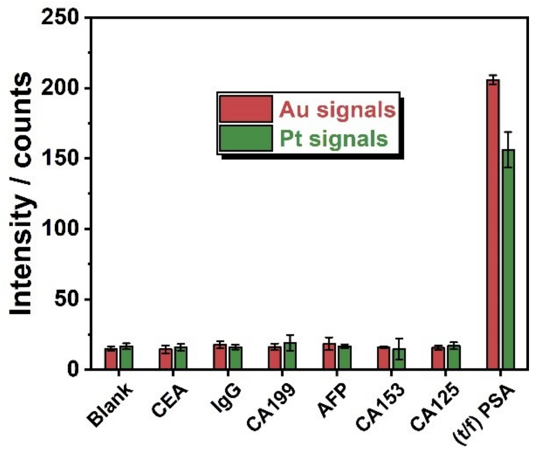

Fig. S14. Selectivity of SMB nanoplatform.

Fig. S15: ROC Curves for Prostate Disease Differentiation

Predictive prognostic values of fPSA (Green), tPSA (Red) and Pt/Au Ratio (Blue) using ROC curves in differentiation of healthy, BPH and cancer groups.

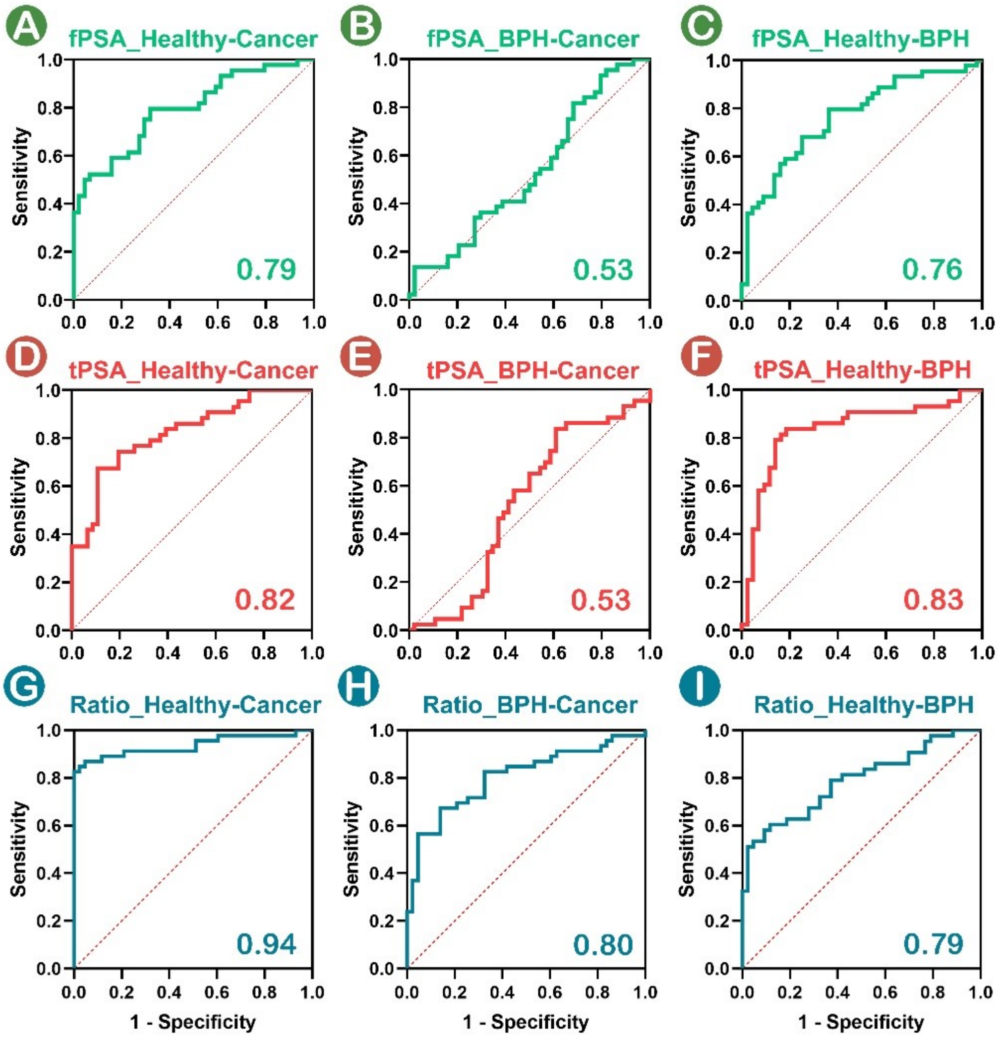

Fig. S15. ROC curves of fPSA, tPSA and Pt/Au Ratio in differentiation of healthy, BPH and cancer groups.

References

1. J. Piella, N. G. Bastús and V. Puntes, *Chemistry of Materials*, 2016, **28**, 1066-1075.
2. N. G. Bastus, J. Comenge and V. Puntes, *Langmuir*, 2011, **27**, 11098-11105.
3. N. C. Bigall, T. Härtling, M. Klose, P. Simon, L. M. Eng and A. Eychmüller, *Nano Letters*, 2008, **8**, 4588-4592.
4. Z. Huang, Z. Li, M. Jiang, R. Liu and Y. Lv, *Anal Chem*, 2020, **92**, 16105-16112.
5. K. Jung, U. Elgeti, M. Lein, B. Brux, P. Sinha, B. Rudolph, S. Hauptmann, D. Schnorr and S. A. Loening, *Clinical Chemistry*, 2000, **46**, 55-62.
6. K. Mitrunen, K. Pettersson, T. Piironen, T. Björk, H. Lilja and T. Lövgren, *Clinical Chemistry*, 1995, **41**, 1115-1120.
7. T. Kokko, T. Liljenbäck, M. T. Peltola, L. Kokko and T. Soukka, *Analytical Chemistry*, 2008, **80**, 9763-9768.
8. Z. Cheng, N. Choi, R. Wang, S. Lee, K. C. Moon, S. Y. Yoon, L. Chen and J. Choo, *ACS Nano*, 2017, **11**, 4926-4933.
9. Z. Rong, Z. Bai, J. Li, H. Tang, T. Shen, Q. Wang, C. Wang, R. Xiao and S. Wang, *Biosens Bioelectron*, 2019, **145**, 111719.
10. V. Escamilla-Gomez, D. Hernandez-Santos, M. B. Gonzalez-Garcia, J. M. Pingarron-Carrazon and A. Costa-Garcia, *Biosens Bioelectron*, 2009, **24**, 2678-2683.
11. R. Gao, Z. Cheng, X. Wang, L. Yu, Z. Guo, G. Zhao and J. Choo, *Biosens Bioelectron*, 2018, **119**, 126-133.
12. Z. Jiang, Y. Qin, Z. Peng, S. Chen, S. Chen, C. Deng and J. Xiang, *Biosens Bioelectron*, 2014, **62**, 268-273.
13. A. Liu, F. Zhao, Y. Zhao, L. Shangguan and S. Liu, *Biosens Bioelectron*, 2016, **81**, 97-102.
14. L. Zhao, D. Wang, G. Shi and L. Lin, *Luminescence*, 2017, **32**, 1547-1553.
15. C. Xie and G. Wang, *J Clin Lab Anal*, 2011, **25**, 37-42.
16. T. Steuber, A. J. Vickers, A. M. Serio, V. Vaisanen, A. Haese, K. Pettersson, J. A. Eastham, P. T. Scardino, H. Huland and H. Lilja, *Clin Chem*, 2007, **53**, 233-240.
17. B. Zhou, J. Zhang, Z. Lv, J. Fan, Y. Zhang and B. Huang, *J Clin Lab Anal*, 2017, **31**.
18. D. Cao, C.-Y. Li, C.-B. Qi, H.-L. Chen, D.-W. Pang and H.-W. Tang, *Sensors and Actuators B: Chemical*, 2018, **269**, 143-150.
19. G. Wu, R. H. Datar, K. M. Hansen, T. Thundat, R. J. Cote and A. Majumdar, *Nature Biotechnology*, 2001, **19**, 856-860.
20. S. Sharma, J. Zapatero-Rodriguez and R. O'Kennedy, *Biotechnol Adv*, 2017, **35**, 135-149.
21. X. Liu, M. Atwater, J. Wang and Q. Huo, *Colloids Surf B Biointerfaces*, 2007, **58**, 3-7.
